# It is time to act on dementia

**DOI:** 10.4103/0972-2327.74244

**Published:** 2010-12

**Authors:** Sanjeev V. Thomas

**Affiliations:** Editor, Annals of Indian Academy of Neurology, Department of Neurology, Sree Chitra Tirunal Institute for Medical Sciences and Technology, Trivandrum - 695011, Kerala, India. E-mail: sanjeev.v.thomas@gmail.com

It was estimated that there were 24 million people with dementia in the world in 2005 and this figure is expected to double every 20 years.[1] About 60% of them live in developing countries. This proportion is likely to reach 75% in the next two decades. The disease burden of dementia is expected to rise more in developing countries. The economic burden of care for people with dementia is 1% of the global Gross Domestic Product. Care mechanisms for persons with dementia are rapidly changing in developing countries. Traditionally, elderly and persons with cognitive impairment were looked after at home by their family members, particularly women in the household. In India and several other developing countries, more women are now attaining higher education and getting employed. There is increasing migration of young people to more developed countries. The smaller family concept is leading to fewer persons at home to look after elderly people. As a result of all these influences, the traditional system of domiciliary care for persons with dementia is becoming more difficult. At the same time, suitable and acceptable alternate systems to attend to them are not well established in these countries. This year, the theme for the world Alzheimer’s Day was “Dementia: It is time to act”. This theme draws the attention of the world to the urgent need to focus on the actions required from all quarters – public, administrators, professionals and care givers alike – to ameliorate the burden due to dementia.

This supplement focuses attention on the clinical aspects of dementia and its management. I congratulate Dr. Mathuranath for bringing together a host of contributors to write practically important articles for this supplement. He had put in tremendous effort to bring out this supplement. I hope that this supplement will make an excellent learning material for our esteemed readers.
